# Phytonanotherapy for the Treatment of Metabolic Dysfunction-Associated Steatotic Liver Disease

**DOI:** 10.3390/ijms25115571

**Published:** 2024-05-21

**Authors:** Livhuwani P. Nendouvhada, Nicole R. S. Sibuyi, Adewale O. Fadaka, Samantha Meyer, Abram M. Madiehe, Mervin Meyer, Kwazikwakhe B. Gabuza

**Affiliations:** 1 Department of Science and Innovation/Mintek Nanotechnology Innovation Centre, Biolabels Research Node, Department of Biotechnology, University of the Western Cape, Bellville 7535, South Africaafadaka@uwc.ac.za (A.O.F.); memeyer@uwc.ac.za (M.M.); 2 Biomedical Research and Innovation Platform, South African Medical Research Council, Tygerberg 7505, South Africa; 3 Health Platform, Advanced Materials Division, Mintek, Randburg 2194, South Africa; 4 Department of Biomedical Sciences, Faculty of Health and Wellness Sciences, Cape Peninsula University of Technology, Bellville 7535, South Africa; meyers@cput.ac.za

**Keywords:** green nanotechnology, metal-based nanoparticles, metabolic dysfunction-associated steatotic liver disease, metabolic dysfunction-associated steatohepatitis, obesity, phytotherapy

## Abstract

Metabolic dysfunction-associated steatotic liver disease (MASLD), previously known as nonalcoholic fatty liver disease, is a steatotic liver disease associated with metabolic syndrome (MetS), especially obesity, hypertension, diabetes, hyperlipidemia, and hypertriglyceridemia. MASLD in 43–44% of patients can progress to metabolic dysfunction-associated steatohepatitis (MASH), and 7–30% of these cases will progress to liver scarring (cirrhosis). To date, the mechanism of MASLD and its progression is not completely understood and there were no therapeutic strategies specifically tailored for MASLD/MASH until March 2024. The conventional antiobesity and antidiabetic pharmacological approaches used to reduce the progression of MASLD demonstrated favorable peripheral outcomes but insignificant effects on liver histology. Alternatively, phyto-synthesized metal-based nanoparticles (MNPs) are now being explored in the treatment of various liver diseases due to their unique bioactivities and reduced bystander effects. Although phytonanotherapy has not been explored in the clinical treatment of MASLD/MASH, MNPs such as gold NPs (AuNPs) and silver NPs (AgNPs) have been reported to improve metabolic processes by reducing blood glucose levels, body fat, and inflammation. Therefore, these actions suggest that MNPs can potentially be used in the treatment of MASLD/MASH and related metabolic diseases. Further studies are warranted to investigate the feasibility and efficacy of phytonanomedicine before clinical application.

## 1. Introduction

MASLD and related liver diseases are increasing worldwide and have become a major public health concern [1]. The pathogenesis of MASLD is still unclear but has been associated with metabolic syndrome (MetS), mainly obesity and diabetes. Hence, antiobesity and antidiabetic drugs are currently used for the management and prevention of MASLD and its progression. Although these drugs can delay the effects of MASLD, they are ineffective at completely reversing or preventing its progression. Additionally, adverse effects, such as an increased risk of heart attack, can occur over time [2], pointing out the urgency of developing strategies with sustainable therapeutic effects MASLD can progress to MASH and cirrhosis, which could in turn lead to end-stage liver disease that will eventually require liver transplantation [3]. To date, the mechanism of MASLD is not completely understood [4] and a treatment is still elusive. There is a renewed interest in plant-based therapy (phytotherapy) due to its long history in folk medicine. Phytotherapy has been shown to be safe and might counteract the side effects of conventional therapies [5]. Phytotherapy has been previously examined for the treatment of MASLD and demonstrated promising outcomes in human clinical trials investigating their impact on MASLD and its associated diseases. However, the insoluble nature of bioactive phytochemicals results in decreased bioavailability and bystander toxicity [6]. The problems associated with plant-based treatments can be overcome with green nanotechnology [7]. Plants contain bioactive components with bioreducing capabilities that have been explored in the synthesis of various MNPs [8]. In this review, the properties of some of the green synthesized MNPs that are beneficial for liver diseases are discussed to show their potential use in the treatment of MASLD/MASH.

## 2. Epidemiology and Pathophysiology of MASLD

MASLD is the most common liver disease that has been reported to affect a quarter of the world’s population [9]. The latest estimate of global prevalence of MASLD in adults was around 30% [10]. The breakdown was as follows: North America and Australia: 32.1%; Asia Pacific region: 28%; Western Europe: 33.1%; South-East Asia: 29.7%; East Asia: 33.8%; South Asia: 33.8%; Latin America: 44%; and the Middle East and North Africa: 36.5% [11]. Data on the prevalence of MASLD in Africa are scarce, but the overall prevalence is estimated to be 13.5% [12]. MASLD is recognized as the hepatic manifestation of MetS [13] and is mostly prevalent in obese people, accounting for 60–95% of the cases [14]. Abdominal obesity is the fundamental pathophysiological factor for metabolic diseases [15], although visceral fat was also associated with the development of MASLD in a 2017 cohort study that lasted 4.4 years with an adjusted hazard ratio of 2.23 (95% CI: 1.28–3.89) [16]. About 69% of patients were also diagnosed with hyperlipidemia, 39% had hypertension, and 22% had diabetes [11]. 

Type 2 diabetes (T2D) has a two-way road with MASLD: the identification of one entity in patients can predispose patients to the other, and treatment can significantly reduce the burden of both diseases. It was projected that T2D would be present in 23% of patients with MASLD and 44% of patients with MASH, which is much higher than the prevalence of diabetes in the general population (8.5%) [11]. MASLD raises the likelihood of developing T2D and speeds up the onset of its consequences [17]. The incidence of MASH is two- to three-fold higher in patients with T2D [18], with 17.7% experiencing advanced liver fibrosis [19] and 7.1% experiencing advanced cirrhosis. In fact, 30–40% of patients with T2D have advanced fibrosis and up to 80% of them have MASH [11]. MASLD and T2D were the first two prevalent variables found in patients with hepatocellular carcinoma (HCC), and T2D alone can double or triple the chances of developing HCC [20]. 

The pathophysiology of MASLD involves numerous factors; diet, hormones, and genes may all have a role in its emergence [21]. It is widely known that the development of steatosis is related to the accumulation of liver fat and insulin resistance (IR), with IR believed to be the primary pathogenic cause of liver steatosis. The ‘two-hit theory’ is a popular paradigm to explain the pathogenesis of MASLD and its progression to MASH. The ‘first hit’ is related to the build-up of triglycerides in the liver, which then makes the liver susceptible to numerous factors that eventually result in the ‘second hit’ which encourages fibrosis, inflammation, and liver damage. Adipokines, pro-inflammatory cytokines, mitochondrial dysfunction, oxidative stress, and subsequent lipid peroxidation are all involved in this process [22] and are known to cause MASLD to proceed to serious liver damage [23]. 

**Figure 1:**
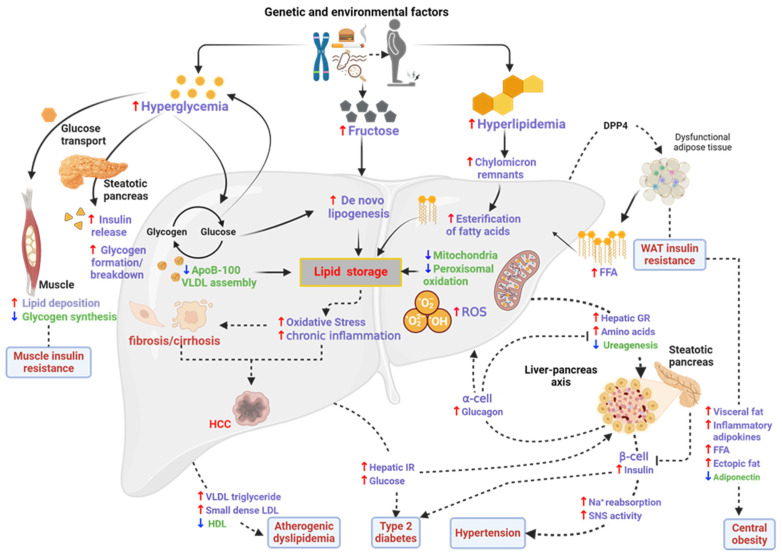
Pathogenesis and progression of MASLD. Obesity causes dysfunction in WAT, leading to an influx of free fatty acids (FFAs) into the liver. IR, glucagon resistance (GR), inflammation, and lipogenesis ensues, which are preceded by features of MetS (dyslipidemia diabetes and hypertension). DPP4—dipeptidyl peptidase 4; AAs—amino acids; AT—adipose tissue; HDL—high-density lipoprotein; LDL—low-density lipoprotein; SAT—subcutaneous adipose tissue; SNS—sympathetic nervous system; VAT—visceral adipose tissue; VLDL—very low-density lipoprotein. Adapted with permission from BMC [25]. Image was drawn using Biorender.com.

Oxidative stress is the starting point for the evolution of steatosis to steatohepatitis. Adipose tissue, particularly visceral adiposity, which is now recognized as an endocrine, paracrine, and autocrine organ, has also been shown to play significant pathogenic roles [24]. The gut–liver axis was also shown to play a crucial role in the development of MASLD. Furthermore, the role of inflammation and high-sugar diets in the etiology of MASLD has been stressed over the past 10 years. Genetic predisposition is one of the main risk factors; several gene polymorphisms have been identified that could affect the way lipids and sugars are metabolized in the liver and other tissues, including adipose tissue [23]. The pathogenesis of MASLD/MASH is a complex process that has many unanswered questions and cannot be explained by the ‘two-hit theory’ alone [25]. The most recent evidence points to the complexity of the disease, which cannot be explained by the accumulation of fat that triggers IR, which is followed by inflammation. There are many factors that are associated with the pathogenesis of MASLD/MASH and can be best described by the ‘multiple-hit theory’ that shows the involvement of many different factors and pathways, as highlighted by Figure 1 [4]. According to the multiple-hit theory, environmental (poor dietary) and genetic factors contribute to the development of IR, obesity, and changes in the gut microbiome. IR is one of the major risk factors for the development of MASLD/MASH, resulting in increased liver de novo lipogenesis and impaired lipolysis. As a result, IR encourages adipose tissue dysfunction, resulting in the altered synthesis and secretion of adipokines and inflammatory cytokines [26]. The details of the “multiple-hit theory” are reviewed elsewhere [25,27].

### 2.1. Current Therapies Used for MASLD 

MASLD and its related diseases have placed a considerable burden on the public health system; yet, until recently, there were no drugs specifically licensed by the Food and Drug Administration for their treatment. Patients with MASH are first treated with dietary adjustments and lifestyle improvements. Many patients are unable to reduce their bodyweight with lifestyle modification alone; therefore, it is always combined with pharmacological interventions [2]. In severe cases, a liver transplant is recommended.

#### 2.1.1. Lifestyle Interventions for MASLD

For all patients with MASLD, lifestyle changes that emphasize 10% weight loss and increased physical activity are essential. Weight loss through lifestyle modifications reduces the cardiovascular risk in patients, improves steatosis, and presumably reduces liver inflammation and hepatocellular injury. In a randomized controlled trial, a low calorie diet accompanied with moderate (200 min/week) physical activity for 48 weeks resulted in an overall bodyweight loss of 9.3%, reduced inflammation, and reduced effects from steatosis on liver biopsy [28].

One of the main pathophysiological mechanisms underlying MASLD is IR, which is reversed by aerobic exercise because it enhances skeletal muscle insulin sensitivity [18]. Moderate and high intensity training all resulted in better liver enzyme functions and reduced steatosis [29]. In sedentary persons with MASLD, Hallsworth et al. found that 8 weeks of resistance training led to improvements in IR and liver lipids were reduced by 13% [30]. Aerobic and resistance exercise for 4 months in randomized controlled trials in inactive people with T2D and MASLD improved insulin sensitivity, and reduced liver steatosis and the hepatic fat content [31]. The recommendation to start physical activity and regular exercise should be given to all patients with MASLD; at least 30 min of brisk activity five times a week may be helpful in its treatment [32]. In parallel to exercise, patients must follow a calorie restriction diet to lose 0.5 to 1 kg per week until they reach their target weight. In general, it is recommended to avoid carbohydrates, saturated fats, and sugar-sweetened beverages [33]. In non-diabetic patients with MASLD, Ryan et al. compared a Mediterranean diet (rich in monounsaturated fatty acids) with a low-fat and high-carbohydrate diet. The two diets did not vary in terms of mean weight loss, but the Mediterranean diet significantly reduced liver steatosis and improved insulin sensitivity [34].

Dietary omega-3 polyunsaturated fatty acids (n-3 PUFAs) have recently attracted attention for the management of MASLD; n-3 PUFAs can reduce liver fat, although it has no significant impact on alanine transaminase (ALT) levels [35]. Dietary supplementation with fish oil can provide patients with MASLD an alternative treatment, although more research is required. In terms of weight loss (5.6 vs. 0.6 kg) and achieving remission of MASLD (64 vs. 20%), dietitian-led lifestyle interventions over 12 months outperformed standard care [36].

#### 2.1.2. Pharmacotherapy for MASLD/MASH

**Figure 2:**
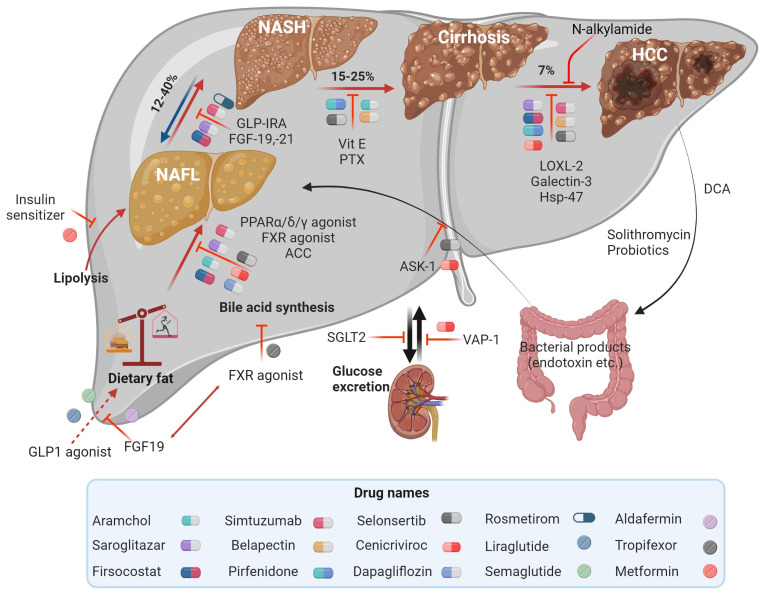
Therapeutic targets in MASLD and MASH involved in metabolism to decrease liver fat deposition or apoptosis and fibrosis. Adapted with permission from Springer [39]. Image drawn using Biorender.com.

Several therapeutic strategies for diabetes and obesity have been shown to improve quality of life and delay MASLD progression [37]. The most significant predictor of liver-related mortality in patients with MASLD is the presence of severe fibrosis (stage ≥ 2) [38]. Therefore, pharmacotherapies are only considered for MASH patients with stage ≥ 2 fibrosis or early stage fibrosis with a high risk of progression [2], with a primary focus on improving liver functions [11]. Some of these drugs are highlighted in Figure 2; the drugs are targeted at various stages of the disease to restore fat and glucose metabolism.

Antidiabetic drugs (pioglitazone, liraglutide, and exenatide) are effective in reducing liver fibrosis [40], while lipid lowering agents (statins or ezetimibe) can reduce the risk of cardiovascular events in patients with MASLD and dyslipidemia. However, these medications have little to no effect on the histology of the liver [41]. The effects of ezetimibe on liver steatosis remain uncertain but it was reported to reduce serum liver enzymes and hepatic steatosis [42]. Antifibrotic and anti-inflammatory compounds have been taken into consideration for the treatment of advanced MASH. Farnesoid X receptor (FXR) agonists also have beneficial health effects in this regard. Obeticholic acid (OCA) reached phase 3 clinical trials for the treatment of MASLD with T2D or MASH, where it improved insulin sensitivity, glucose homeostasis, and lipid metabolism, and exerted anti-inflammatory and antifibrotic actions in the liver. The trials also evaluated the effects of synthetic non-bile acid FXR agonists, which have the potential to deliver positive metabolic effects without worsening adverse effects such as gastrointestinal problems, pruritus, fatigue, and headache [43]. Antiobesity drugs are used to help patients lose weight but their specific effect on MASH is not yet clear. Drugs such as naltrexone/bupropion, phentermine/topiramate, or lorcaserin may have an indirect effect on MASLD progression [44].

The antifibrotic drug emricasan, a caspase inhibitor that improved fibrosis in mouse models of MASH, was investigated for its efficacy in MASH (stages 1–3), including those with cirrhosis and severe portal hypertension [45]. Pentoxifylline (PTX) dropped MASLD activity by a score of 2 points in 38.5% of patients after one year of treatment (versus 13.8% with placebo). Steatosis, inflammation, and fibrosis were all significantly reduced by PTX treatment [46]. PTX also improved liver histological findings, such as lobular inflammation, without changing lipid profiles. However, the original efficacy of the drug to treat the effects of brain stroke was deemed insufficient and is no longer commercially accessible in Japan [39]. There are a few studies that showed that angiotensinogen receptor blockers could have antifibrotic effects in MASH; clinical trials are required to confirm this [47].

Antioxidant-rich vitamins, such as vitamins E and C, were shown to improve the management of MASLD by reducing serum ALT and AST, as well as lipoatrophy and lobular hepatitis [37]. Vitamin E is a well-known free radical scavenger and was shown to reduce serum transaminase activities and transforming growth factor beta-1 levels in adult MASH patients [40]. In non-diabetic patients with MASH, vitamin E improved their liver histology but has not yet been tested in patients with diabetes or cirrhosis. Vitamin E in adult [4] and pediatric MASH patients [48] reduced steatohepatitis. However, there are some safety concerns, as large doses could lead to an increased risk of hemorrhagic stroke [48] and prostate cancer. Presently, only select patients with advanced precirrhotic MASH who have not responded to lifestyle treatments are recommended for vitamin E treatment [37].

Glutathione (GSH; L-glutamyl-L-cysteinyl-glycine), a tripeptide that is present in the human body, has an antioxidant effect. In a pilot study, the oral administration of GSH reduced ALT levels and hepatic steatosis in patients [49]. Ursodeoxycholic acid (UDCA), an antioxidant agent, did not show the desired effects on liver histology in MASH; however, some studies have shown that in higher doses, it might have favorable effects [50]. Several innovative drugs targeting various phases of the disease are still in development; their therapeutic effects are summarized in Table 1. However, most drugs, such as OCA, selonsertib, elafibranor, and cenicriviroc, did not pass phase 3 trials and did not receive FDA approval for clinical use due to safety issues and their low efficacy [51]. 

Pegozafermin and resmetirom are currently the promising anti-MASLD/MASH therapy with clinically beneficial effects. Pegozafermin, a fibroblast growth factor 21 (FGF21) analogue, improved fibrosis without worsening the disease in MASH patients treated with three doses (15, 30, and 44 mg) for 24 months. The ENLIVEN phase 2b trial demonstrated reduced liver fat, adiponectin levels, and triglyceride levels, and improved MASH biomarkers (AST, ALT). Adverse effects were higher in weekly treatments (95% for 15 mg, 85% for 30 mg) and reduced in the bi-weekly treatment (67% for 44 mg). Acute pancreatitis and gallbladder sludge was observed in one patient on 44 mg pegozafermin. Pegozafermin is set for a phase 3 clinical trial (NCT04929483) to investigate the drug’s safety, efficacy, and tolerability for a longer duration [52]. A breakthrough in MASLD/MASH therapy was reported in March 2024 when Resmetirom or Rezdiffra was approved by the US Food and Drug Administration (FDA) for non-cirrhotic MASH in adults with moderate to advanced fibrosis [53]. Resmetirom is administered through the oral route to reduce liver fat by activating the thyroid hormone receptor. In a phase 3 clinical trial, treatment with a single dose of resmetirom at 80 or 100 mg for 54 weeks did not worsen the fibrosis or MASLD activity score. The two doses showed adverse effects in 90% of the patients; nausea and diarrhea were more common in treated patients compared to those receiving the placebo [54]. An ongoing postapproval study will investigate the clinical benefit of the two doses of Resmetirom for 54 months. The limiting factors of the drug include liver toxicity and gall bladder side effects [53]. 

#### 2.1.3. Liver Transplantation

MASH cirrhosis is a frequent reason for liver transplantation, where the patient and graft survival rates are overall excellent [55]. Patients who underwent liver transplantation for cryptogenic cirrhosis discovered that while all grafts had evidence of steatosis 5 years after the transplant (compared to 25% in age- and sex-matched controls with primary biliary cirrhosis and primary sclerosing cholangitis), only 11% of these patients progressed to steatohepatitis [56]. Another study that looked at 98 patients who had their livers transplanted due to MASH cirrhosis showed that 25% of them had MASH and that 70% of them had recurrent steatosis, but none of them had graft failure or needed another transplant after 3 years. To reduce the risk of cardiovascular-related mortality, it is essential to control the cardiovascular risk factors after the transplant [57].

## 3. Phytotherapy as an Alternative Treatment for MASLD

The relevant targets that are important for the treatment of MASLD/MASH were guided by conventional therapy borrowed from antiobesity, antidiabetic, antifibrotic, and anti-inflammatory drugs. Although some are effective to some extent, most of these drugs have no effect on liver histology and more clinical trials are still required to prove their safety [40]. Due to the side effects associated with these treatments, such as increased risk of cardiovascular disorders, some of these treatments have been discontinued or withdrawn from the market. Therefore, it is no longer possible to overlook the need for an efficient treatment that targets the pathophysiological mechanisms of MASLD/MASH to develop safer and disease-specific therapies. 

Plants have played a significant role for centuries in the treatment of various diseases as they contain phytoconstituents with biologically relevant properties. To date, they have been explored in the search for novel bioactive compounds that can alleviate the burden of infectious and chronic diseases. Scientific evidence is continuously emerging to support the beneficial health effects of plant-based therapy against various diseases, including liver diseases [58]. Basically, plants that have anti-inflammatory, antioxidant, and lipid and glucose metabolism effects are crucial in the treatment of liver diseases, as reviewed elsewhere [59]. Several plants have been shown to contain beneficial health effects on MetS, and are used in traditional medicine to treat diabetes, obesity, and cardiovascular diseases, among others. Medicinal plants used in the treatment of MetS have hepatoprotective activity and are capable of reducing liver fat. Some examples of plants with the potential to ameliorate liver diseases and possibly MASLD/NASH are summarized in Table 2. 

African Cucumis (*Cucumia africanus*) is traditionally used to treat viral hepatitis, gonorrhea, inflammation, pain, and skin problems. The benefits of African Cucumis are unmatched by any other herbal remedy used for liver disorders and diseases; as a result, it is recommended for people who regularly consume unhealthy fatty meals and alcohol. Some of its medicinal properties are similar to those of *Pedicellus Melo* (Tian Gua Di, China), which is used to treat a variety of conditions, including hepatocirrhosis, liver cancer, and acute and chronic viral hepatitis [60]. Guava (*Psidium guajava linn*.) fruit, leaf, stem bark, or root extracts are widely used in folk medicine as antioxidant, antispasmodic, antiallergy, anti-inflammatory, and antidiabetic agents [65]. Marigold flower extract was shown to have protective effects against hepatotoxicity in streptozotocin-induced diabetic rats, as evidenced by alterations in blood biochemical markers such as glucose, ALT, aspartate aminotransferase (AST), and lactate dehydrogenase activity [62]. Milk thistle (*Silybum marianum*) has been used to treat liver, kidney, and gallbladder problems. It contains compounds that protect the liver from toxins and medications such as acetaminophen (Tylenol), which can harm the liver in large amounts. This is attributed to the presence of a flavonoid called silymarin. Milk thistle increases the survival rates in people with cirrhosis or chronic hepatitis and enhances liver function [63]. Dandelions (*Taraxacum officinale*) are a great source of vitamins A, B, C, and D, as well as minerals such as iron, potassium, and zinc. The roots and leaves of dandelion have traditionally been used to treat liver problems. Native Americans used dandelion to treat heartburn, edema, skin problems, renal disease, and upset stomach by boiling it in water and consuming it [64]. Some of the effects of these plants’ active compounds in clinical trials and their mechanisms against MASLD and its related diseases were reviewed by Rizzo et al. [66] and Yan et al. [6]. There are several ongoing and completed clinical trials using silymarin, berberine, resveratrol, Zhenzhu Tiaozhi, anthocyanin, bayberry juice, curcumin, protandim, siliphos, and Zataria multiflora Boiss. Berberine, silymarin, resveratrol, and curcumin are in advanced trials and have been reported to target multiple molecular mechanisms [6]. 

Traditional medicine has always used a holistic approach, where a whole plant extract was used in the treatment of diseases. A holistic approach may have a synergistic effect that increases the solubility, availability, and biocompatibility of the bioactive compound, which is lacking in individual phytochemicals. This approach is practiced in Chinese herbal formulations and has been demonstrated to be more effective in tackling the multifaceted nature of MASLD/MASH. Chinese herbs used in holistic approaches include Shanzha (Crataegi Fructus), Danshen (Salviae Miltiorrhizae radix et Rhizoma), Fuling (Poria), Zexie (Alismatis rhizome, Chaihu (Bupleuri Radix), Juemingzi (Cassiae Semen), Yujin (Curcumae Radix), and Baizhu (Atractylodes macrocephala Koidz.). These are formulated to also treat syndromes that are associated with or caused by MASLD/MASH, i.e., herbs that tonify the spleen, regulate qi, eliminate and dehumidify phlegm, activate blood, and clear the liver [67]. Plant extracts have similar mechanisms to currently used drugs; their advantage in treating MASLD/MASH is that they can target multiple molecular mechanisms of the disease. For example, Shanzha contains several bioactive compounds (chlorogenic acid and hyperoside oleanolic acid) that collectively or individually have hepatoprotective activities. Chlorogenic acid in MASH models increases adiponectin expression, prevents liver injury, and reduces insulin resistance; oleanolic acid, through the adenosine monophosphate-activated protein kinase (AMPK) pathway, is able to inhibit lipogenesis and enhance insulin sensitivity. Thus, Shanzha can potentially target several pathogenic pathways through a single treatment, e.g., the c-Jun N-terminal kinase (through chlorogenic acid) and AMPK (through oleanolic acid) pathways [67]. Recent studies showed that the properties of the above phytoconstituents can be enhanced and their limitations can be overcome when they are used as reducing agents in the synthesis of MNPs. 

## 4. Phytonanotherapy for MASLD/MASH

Green nanotechnology is an environmentally friendly and economical process adopted to produce nanomaterials (in the size range of 1 to 100 nm) using natural products from plants or microorganisms. Green synthesis can counteract the unanticipated hazardous effects of the traditional (chemical reduction) method of synthesis [68]. The general toxicity of the NPs produced by chemical reducing agents raises health concerns. Plant-mediated synthesis of NPs is preferred over microbial synthesis because it has a faster production rate, is easy to scale up, and is more affordable [69]. Green synthesis offers many advantages that can improve existing phytomedicines, with the potential to enhance the bioactivities of phytochemicals involved in the bioreduction and stabilization of NPs [7].

**Figure 3:**
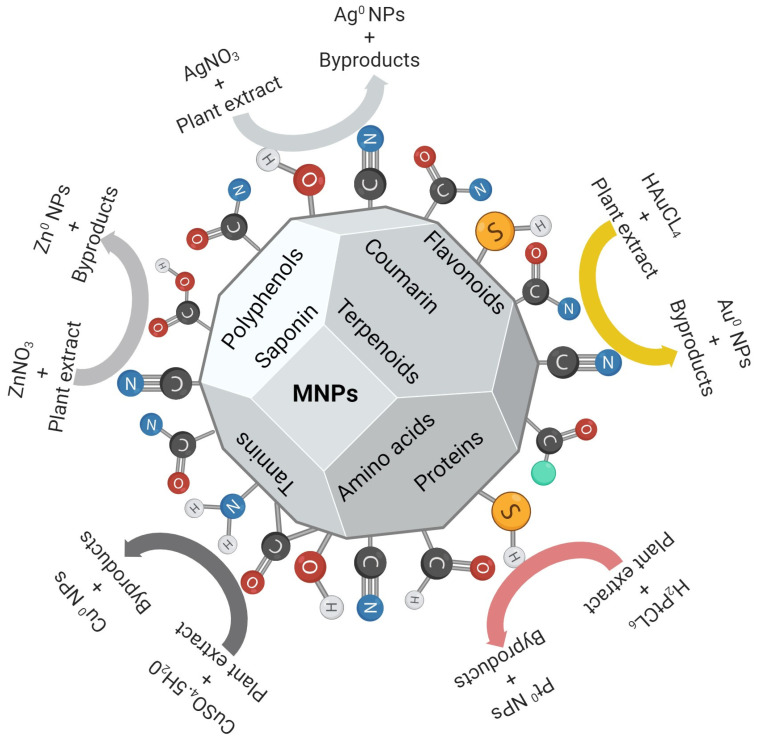
Plant-mediated synthesis of various MNPs. Different phytochemicals contain functional groups that can reduce metal precursors into MNPs, and some serve as stabilization agents. Adapted with permission from MDPI [72]. Image was drawn using Biorender.com.

Plants are used as environmentally benign biological factories for green-chemistry-based processes. Plant extracts from various parts, such as leaves, roots, stems, barks, etc., are used to economically produce bioactive MNPs [8]. Alkaloids, proteins, phenolic acids, sugars, terpenoids, and polyphenols are some of the bioactive phytochemicals that have been shown to play a significant role in the reduction and stabilization of various metal precursors (Figure 3). The synthesis method using plant extracts was reviewed by Aboyewa et al. Silver NPs (AgNPs) and gold NPs (AuNPs) are by far the most researched MNPs [8]. In the nanometer size range, MNPs have unique properties compared to their bulk counterparts, and these properties have been exploited for various bioapplications. NPs in the size range of 1–500 nm are able to penetrate and pass through biological barriers [70] including the blood–brain barrier. Their small size, coupled with other properties such as surface composition, charge, and shape, amongst others, controls how the NPs interact with and are taken up by cells [71].

Therapeutic benefits of MNPs have been reported in various disease models, including obesity, diabetes, and MASLD models. Chemically synthesized NPs showed contradictory effects in in vitro and in vivo models of MASLD/MASH. In an in vitro steatotic model, AgNPs, titanium dioxide NPs (TiO_2_ NPs), and zirconium dioxide NPs were cytotoxic to HepG2 cells at ≥10, ≥20, and ≥200 µg/mL, respectively. The MNPs were more cytotoxic toward steatotic HepG2 cells, and the most potent MNPs (AgNPs and TiO_2_ NPs) did not have significant effects on the total cholesterol and triacylglycerol contents. The AgNPs altered the expression of genes involved in fatty acid metabolism and antioxidant responses [73]. On the contrary, a negative regulatory effect of AgNPs was reported in a high-fat diet-induced MASLD mouse model. A single dose of AgNPs at 0.5–12.5 mg/kg bodyweight worsened liver function and histology within 7 days, which progressed from MASLD to MASH. Markers of the disease were increased; these included lipid deposition in the liver, oxidative stress, inflammation, and plasma levels of ALT and AST. The AgNP-induced hepatotoxicity was accompanied by an up-regulation of genes involved in liver lipogenesis [74]. These effects were consistent in both intravenously [74] and orally administered AgNPs [75]. The prospective use of MNP-based therapy for the treatment of MASLD/MASH is motivated by the hepatoprotective effects demonstrated by the nanomaterials. AgNPs (3–5 nm) could restore liver function after acetaminophen-induced liver injury in Wistar rats. AgNPs also restored the levels of AST, ALT, alkaline phosphatase, lactate dehydrogenase, cholesterol, triglyceride, and markers of oxidative stress (lipid peroxidation, reduced glutathione, superoxide dismutase, an d catalase) and liver physiology in a dose-dependent manner [76]. Daily intake of alcohol supplemented with AuNPs (30–50 nm) for 10 weeks protected rats from liver damage [77]. In another study, nanogold (2–5 nm) in water was able to reverse the effects of carbon tetrachloride-induced hepatic injury in rats [78]. Oral gavage of 7.4 nm AuNPs an hour before the animals were exposed to ethanol and methamphetamine protected the rats from liver injury. This was attributed to the antioxidant, anti-inflammatory, and antifibrotic effects of the AuNPs [79]; these effects were also observed in other inflammatory diseases. In a lipopolysaccharide (LPS)-induced acute inflammation model, 20 nm citrate AuNPs reduced the plasma levels of liver injury markers, leukocyte count, and pro-inflammatory cytokines, and increased anti-inflammatory cytokines, alveolar septum fibrosis, and oxidative damage [80].

The attributes described above have contributed significantly to MNP-based therapies; however, the use of chemically synthesized MNPs remains a major concern. Several studies showed that the surface modification of MNPs could reduce their toxicity through the conjugation of biomolecules to achieve active targeting or to act as stabilizers to prevent the leaching of metal ions and their toxicity [81]. Chemical conjugation for the synthesis of MNPs can involve many steps which can be avoided using plant extracts, which only require one step. Plant extract-synthesized MNPs also have bioactivities that are crucial for MASLD/MASH; these include lipolytic, antifibrotic, antidiabetic, antioxidant, and anti-inflammatory effects. 

### 4.1. Plant-Based MNPs Alleviate Features of MASLD/MASH

Multiple characteristics can be observed during the development and progression of the disease, and over the years, drugs targeting one or more disease-associated biomarkers have been used for disease management [82]. Similarly, plant-based MNPs that can reduce fat deposition in adipose tissues or the liver, lower blood glucose, improve insulin sensitivity, reduce inflammatory responses, etc., can potentially be used in the treatment of MASLD/MASH [83]. Plant-based MNPs offer many advantages, as one type of NP can have more than one of the activities mentioned above. Furthermore, plant-synthesized MNPs are known to selectively target, penetrate, and reverse the effects on affected cells, and the metal core and phytochemicals on their surface can act synergistically to provide sustainable therapeutic effects [84]. A number of plant species with medicinal properties were reported to synthesize MNPs with some of the properties that are of health benefit to MetS, to name a few, include lipolytic, anti-diabetic, antioxidant, and anti-inflammatory activities (Table 3). Most of these plants contain bioactive metabolites, of which, flavonoids, alkaloids, and phenolics have been associated with the bioreduction, stabilization, and bioactivities of MNPs [8]. The immunomodulatory, cardioprotective, hepatoprotective, and cancer prevention effects of these phytochemicals have been praised for centuries and continue to drive the discovery of novel drugs [85].

Biogenic ZnO NPs, AgNPs, and AuNPs are among the MNPs that can reverse the effects of MASLD and MASH. These MNPs are perceived to be superior to their chemically synthesized counterparts, especially with regard to biosafety. There are no plant-based MNPs that are specifically used for MASLD and MASH; this review only highlights the ones that have beneficial bioactivities on the symptoms of MASLD and its related diseases.

#### 4.1.1. Antioxidant and Anti-Inflammatory Activities of Plant-Based MNPs

Inflammation is a huge problem for MetS and is associated with the progression of MASLD to MASH. Current anti-inflammatory agents have shown bystander toxicity, and chronic inflammation is a risk factor for arthritis, bowel diseases, asthma, stroke, psoriasis, and atherosclerosis. The anti-inflammatory effects reported for various types of MNPs such as AuNPs, zinc oxide NPs, copper oxide NPs, platinum NPs, ceria NPs [93], and AgNPs [84] could be beneficial in the treatment of inflammatory diseases including MASLD/MASH. MNPs can mimic the catalytic activities of various metabolic enzymes such as peroxidase, oxidase, catalase, and superoxide dismutase [86]. The feasibility of green-synthesized MNPs in the treatment of inflammatory diseases was detailed by Li and co-workers [94]. Using antioxidant-rich bioactive compounds as reducing and stabilizing agents in the synthesis of MNPs often results in synergistic bioactivities (reactive oxygen species-scavenging effects) while ensuring their biocompatibility. 

Plant-synthesized MNPs have been reported to scavenge free radicals and inhibit the activities of metabolic enzymes, including those associated with MetS. ZnO NPs synthesized from *Vitis vinifera* peel extract (Vv-ZnO NPs) had antioxidant activity and were shown to be biocompatible and eco-friendly [86]. *Costus igneus Nak* (Ci)-ZnO NPs had antioxidant and antidiabetic activities; the latter was based on their ability to inhibit the actions of α-amylase and α-glucosidase [88]. These NPs did not have any significant effect on the body weight and blood chemistry of rats that were injected intravenously with 200 ug/mL of *Azadirachta indica* extract-ZnO NPs for 14 days. Liver and kidney function tests and histology also suggested that the NPs were biocompatible [87].

AgNPs are widely used as antimicrobial agents against a broad spectrum of microbes, and they are also recognized for their multifunctional properties. Of interest for liver diseases are their anti-inflammatory and antioxidant activities. Plant-based AgNPs prevent or reduce inflammation by inhibiting or enhancing the expression of pro-inflammatory or anti-inflammatory genes, respectively. In vitro, *C. orbiculata* AgNPs reduced the expression of TNF-α, IL-6, and IL-1β in LPS-differentiated THP-1 macrophages [89]. AgNPs synthesized from garlic (*Allium sativum* L.) inhibited the denaturation of bovine serum albumin; protein denaturation is also involved in inflammation, and any agent that can prevent this process could have anti-inflammatory properties [90]. In vivo, AgNPs from *Viburnum opulus* L. fruit extract reduced pro-inflammatory cytokine production and cyclooxygenase activity in Wistar rats with carrageenan-induced hind paw edema [91].

The anti-inflammatory and immunomodulatory effects of *Hypoxis hemerocallidea* and *Hypoxoside* AuNPs also suggest that these NPs can be used for the treatment of inflammation [92]. More information on various plant-based MNPs with antioxidant activities was given by Khan and colleagues [72]. The consensus is that any compound with antioxidant activity will likely have anti-inflammatory properties and the ability to inhibit metabolic enzymes. All of these MNPs could be useful anti-inflammatory agents in the treatment of MASLD/MASH.

#### 4.1.2. Antiobesity Activity of Plant-Based MNPs

Studies have shown that a reduction in bodyweight by 5–10% can restore metabolic functions in patients with diabetes, obesity, and MASLLD/MASH [95]. Hence, the management of MASLD/MASH focuses on improving body metabolic functions and reducing body weight. Antidiabetic and antiobesity drugs do improve and delay the effects of MASLD [96]. Studies are now also exploring the possibility of plant-based MNPs to reverse obesity and MASLD. Nanotwisted *Smilax glabra*-AuNPs at 50 mg/kg body weight showed activity similar to metformin at 300 mg/kg body weight in obese Wistar rats with streptozotocin-induced diabetes. *Smilax glabra*-AuNPs reduced body weight, improved glucose tolerance, and restored liver and heart functions. These AuNPs showed hepatoprotective and anti-inflammatory effects through reducing liver markers (ALT, AST, and ALP) and pro-inflammatory markers (TNF-α and IL-1β), respectively. The antiobesity effects were validated by the changes observed in the expression of adipokines (resistin, adiponectin, and leptin). There was a decrease in the blood levels of total cholesterol, free fatty acids, triglycerides, and high- and low-density cholesterols, and a reduction in the atherogenic and coronary risk indices. These NPs had an effect comparable to that of a FDA-approved diabetes drug, at a dose that was six times lower [97]. AuNPs synthesized from a *Salacia chinensis* extract also had similar effects on the liver, lipid profile, and pro-inflammatory, adipokine, and biochemical markers in high-fat diet-induced obese rats. Their effects were confirmed to occur via the AMP-activated protein kinase pathway [98]. Copper NPs synthesized from Nigella sativa seed extracts demonstrated potential antiobesity and antidiabetic effects by inhibiting the enzyme activities of lipase and α-amylase, respectively [99].

#### 4.1.3. Antidiabetic Activity of Plant-Based MNPs

In most cases, MetS therapeutic strategies target multiple pathways. Similar effects were also observed with plant-synthesized MNPs. MNPs with antioxidant, anti-inflammatory, and antiobesity effects also demonstrated improved glucose metabolism, suggesting that these NPs might have antidiabetic effects. For example, in addition to the respective antioxidant and antiobesity activities of the *Costus igneus Nak* (Ci)-ZnO NPs [88] and Nigella sativa-copper NPs [99], the NPs showed potential antidiabetic activities by inhibiting α-amylase activity. α-amylase is responsible for the breakdown of carbohydrates and increases blood glucose levels. The antidiabetic effects of *Sambucus nigra* L.-AuNPs [100] and *Gymnema sylvestre* R. Br-AuNPs [101] were accompanied by antioxidant and anti-inflammatory activities in diabetic rats. 

## 5. Future Perspectives 

There are constant improvements in the treatment strategies for chronic diseases, including MetS, but there was still no disease-specific treatment or treatment that is effective in treating MASLD/MASH. Adverse effects are a major limitation for conventional therapies, and this has revived the interest in phytomedicine as a safer alternative. Using green nanotechnology, nanomaterials with unique properties are produced. The ability of plant-based MNPs to restore liver physiology and function suggests that they could have preventive effects for MASLD/MASH and could be used for their treatment. Plant-synthesized MNPs have many properties, including anti-inflammatory, antioxidant, and antiobesity activities, among others, targeting most of the important pathways involved in the development and progression of MASLD. MASLD and MASH as multifactorial diseases will benefit from these multifunctional MNPs, as they can target more than one pathway at a time. 

The mechanisms by which these MNPs induce these activities have not yet been elucidated and warrant confirmatory studies to address the concerns surrounding their clinical applications. One of the major concerns of MNPs is the uncertainty of their fate when used in vivo because they are not biodegradable. Although size, surface charge, and composition also affect how MNPs interact with cellular components and their biodistribution, it should also be noted that the liver is part of the reticulo-endothelial system (RES) [102]. As part of the RES, it might also be challenging to target therapeutic agents, especially MNPs that could appear as foreign materials and be cleared from the body. The best advantage of using plant-based MNPs in the treatment of MASLD and its related diseases will be their ability to confer their activity through different routes, their size-dependent activities, and their easily modifiable surfaces. Changing the size or surface composition will alter their pharmacokinetics, resulting in systems with enhanced circulating times, bioavailability, biocompatibility, and escape from the RES or the phagocytic system [103].

There are several targets that have been identified in pathological livers; some are unique, while others overlap with other metabolic diseases. Using nano-based strategies, targeting these biomolecules has showed promising outcomes for the treatment of liver-related diseases in preclinical studies [104]. While plant-synthesized MNPs are perceived as biocompatible [8], the negative effect that chemically synthesized MNPs have on MASLD and liver-related diseases cannot be ignored [105]. It is therefore imperative to evaluate the type of interactions between the metal core and the phytochemicals on their surface. Are the bonds strong enough to withstand the biological environment and protect the metal ions from leaching? Although, there are currently no plant-based MNPs for the treatment of clinical MASLD and MASH, animal studies suggest that these MNPs are effective in reversing the characteristics of MASLD/MASH and are biocompatible. Most importantly, cMNPs in clinical trials demonstrated promising pharmacokinetics, which might imply that they can be substituted with plant-based MNPs and have enhanced bioactivities. The nano Swarna Bhasma drug (AuNPs synthesized using mango peel mixed with five plant extracts) in combination with doxorubicin or cyclophosphamide improved the drug’s response and safety in breast cancer patients [106]. The plant extract-derived MNPs registered in ClinicalTrials.gov are used as antimicrobial agents in dental care products. Mouthwash containing AuNPs synthesized using pelargonium graveolens leaf extract (NCT05816512) in 7–14 year children suffering from gingivitis reduced plaque, gingival, and calculus indices. The mouthwash was used twice daily for 3 weeks [107]. Thyme and carvacroll AgNPs (NCT04431804) are registered as an antifungal agent for *Aspergillus Fumigatus* isolated from patients in the intensive Care. The results from the trial are still pending [108]. 

A human trial on obese MASLD patients using nano-curcumin (curcumin encapsulated in polylactic-co-glycolic acid NPs) capsules produced by the Exir-Nano-Sina Company proved that nanomaterials can be used to effectively deliver therapeutic agents to pathological livers. Nanocurcumin capsules had beneficial effects on important markers for MASLD: serum glucose, lipids (total cholesterol, triglyceride, high density lipoprotein cholesterol, and low density lipoprotein-cholesterol), nesfatin, inflammatory markers (IL-6, TNF-α), and liver transaminases (ALT, AST) levels [109]. This action might possibly be due to the nanocurcumin capsules’ ability to suppress appetite in obese MASH patients [109]. These studies prove that nano-based strategies have the potential to be developed into a potent anti-MASLD/MASH therapy, which will target multiple targets or mechanisms (highlighted in Figure 2). The multifunctional properties of plant-based MNPs would be instrumental in producing systems that combine the bioactivities of plant extracts and the metal core, resulting in sustainable effects. Investigations are then warranted to determine and ensure the stability of plant-synthesized MNPs and their ability to treat MASLD/MASH. 

## 6. Conclusions

To combat MASLD and its related diseases, more efficient treatment options are required that can be targeted at pathological tissues. Plant-based MNP therapy could improve the safety profile of MASLD treatments and provide sustainable therapeutic effects with reduced adverse effects. It has been a challenge to develop specific therapeutic strategies for MASLD/MASH, mainly due to the physiology of the affected organ (the liver). With the emergence of green nanotechnology, it is possible to develop strategies that can be targeted and accumulated in the liver long enough to impart their activities. This was made possible due to the unique physicochemical properties of MNPs that can be tailored to target specific cell types. Plant-mediated MNPs represent an innovative strategy for MASLD/MASH where phytomedicine and nanotechnology offer synergistic disease-specific treatments that are sustainable. 

## Figures and Tables

**Table 1:** Therapeutic targets for MASLD/MASH. Adapted from [39].

Drug	Mechanism	Primary Outcomes	Disease Stage		
			Steatosis	Hepatitis	Fibrosis
Aramchol	FXR ligand	Improvement in fibrosis without worsening MASH	X		X
Obeticholic acid	FXR agonist	Improvement in histological features with discrete and non-significant reduction in fibrosis	X		
Resmetirom	THRβ agonist	Reduction in liver fat and reduction in noninvasive markers of fibrosis	X		X
Lanifibranor	Pan-PPAR agonist	MASH resolution and fibrosis improvement	X		X
Semaglutide	GLP1 agonist	Improvement in histology in patients with MASH	X		
Elafibranor	PPARα/σ agonist	Did not meet predefined primary end point in phase III trial and discontinued			
Saroglitazar	Peroxisome proliferator-activated receptor α/γ agonist	Improvement of MASH without worsening of fibrosis.	X	X	X
Firsocostat (GS-0976)	Acetyl-CoA carboxylase inhibitor	Improvement in liver fat	X	X	X
Simtuzumab	Lysyl oxidase-like-2 inhibitor	Improvement in fibrosis	X	X	X
Belapectin (GR-MD-02)	Galectin-3 inhibitor	Improvement in hepatic venous pressure gradient		X	X
Pirfenidone	Heat shock protein 47 inhibitor	Improvement in fibrosis		X	X
Selonsertib	Apoptosis signal-regulating kinase-1 inhibitor	Improvements in liver histology	X	X	X
Cenicriviroc	Vascular adhesion protein 1 inhibitor	Attenuation of fibrosis	X		X
Dapagliflozin	Sodium–glucose co-transporter 2 inhibitor	Improvement in ALT levels	X		

Note: X—represent disease stage targeted by the treatment.

**Table 2:** Medicinal plants traditionally used for the treatment of different liver disorders.

Plant	Active Compound	Liver-Related Disease	Mode of Action/Bio-Activities	Refs.
African Cucumis	Cucurbitacins	Viral hepatitis	Promote antiproliferation and cell cycle arrest	[60]
Guava leaves	Terpenoids Flavonoids Tannins	NAFDL	Inhibit oxidative stress	[61]
Marigold flower	Phenolics Flavonoids	Toxic liver disease	Inhibit enzymes such as aldose	[62]
Milk thistle	Flavonoid (silymarin)	Alcoholic hepatitis	Increase liver cell regeneration by enhancement of DNA and RNA synthesis	[63]
Dandelion	Sesquiterpene lactones Taraxasterol	Toxic liver disease	Anti-inflammatory, anti-oxidative, anti-carcinogenic activities	[64]

**Table 3:** Plant-based MNPs with bioactivities that can be beneficial to MASLD/MASH.

Plant Species	Type of MNPs	Activities	Assay	Model	Refs.
*Vitis vinifera* exocarp	ZnO NPs	Antioxidant	2,2-diphenyl-1-picrylhydrazyl (DPPH) assay	In vitro	[86]
		Antioxidant enzyme activity	Catalase (CAT) Glutathione peroxidase (GPx) Reduced glutathione (GSH) Glutathione S transferase (GST)	In vitro	
		Metabolic enzyme activity	Acetylcholine esterase (AChE) α-carboxyl and β-carboxylesterase activity Acid and alkaline phosphatase activity	*Diprion similis Artemia salina*, *Aedes aegypti*	
*Azadirachta indica* (Neem tree or Indian lilac)	ZnO NPs	Antioxidant activity	DPPH assay Phosphomolybdenum complex method Ferric reducing antioxidant power (FRAP) assay Lipid peroxidation inhibition assay (ferric thiocyanate method)	In vitro	[87]
		Enzyme inhibition activity	α-glucosidase inhibition assay Butyryl cholinesterase and AChE assay Lipoxygenase inhibition assay		
*Costus igneus Nak* (insulin-rich) leaf extract	ZnO NPs	Antidiabetic Activity	α-amylase inhibition assay α-glucosidase inhibition assay	In vitro enzymatic assay	[88]
		Antioxidant activity	DPPH Assay	In vitro	
*Cotyledon orbiculata* (*C*. *orbiculata*)	AgNPs	Anti-inflammation activity	IL-1β, IL-6, and TNF-α DuoSet ELISA	THP-1 differentiated macrophages NK cells	[89]
Garlic (*Allium sativum* L.)	AgNPs	Anti-inflammation	Inhibition of bovine serum albumin (BSA) denaturation	In vitro	[90]
*Viburnum Opulus* L. (cranberry bush) fruit extract	AgNPs	Anti-inflammation	*Carrageenan-induced hind paw edema* Inhibition of pro-inflammatory cytokines (IL-1α, IL-1β, IL-6, IL-10, and TNF-α)	Wistar rats	[91]
*Hypoxis* *hemerocallidea*	AuNPs	Anti-inflammation Immunomodulation	IL-1β, IL-6, INF-γ, and TNF-α DuoSet ELISA	THP-1 differentiated macrophages NK cells	[92]
*Hypoxoside*	AuNPs	Anti-inflammation Immunomodulation	IL-1β, IL-6, INF-γ, and TNF-α DuoSet ELISA	THP-1 differentiated macrophages NK cells	[92]

## Data Availability

Data sharing not applicable to this article as no datasets were generated or analyzed during the current study.
